# Transcriptomic, proteomic and biochemical comparison of luminescent and non‐luminescent Keroplatinae larvae (Diptera: Keroplatidae)

**DOI:** 10.1111/imb.70008

**Published:** 2025-08-21

**Authors:** Jaqueline R. Silva, Gabriel F. Pelentir, Danilo T. Amaral, Cassius Stevani, Vadim R. Viviani

**Affiliations:** ^1^ Departamento de Física, Química e Matemática Universidade Federal de São Carlos Sorocaba Brazil; ^2^ Programa de Pós‐Graduação em Biotecnologia, Universidade Federal de São Carlos São Carlos Brazil; ^3^ Laboratório de bioinformática para bioprospecção e mineração de dados ômicos, Centro de Ciências Naturais e Humanas, Universidade Federal do ABC (UFABC) São Paulo Brazil; ^4^ Departamento de Química Fundamental Instituto de Química, Universidade de São Paulo São Paulo Brazil

**Keywords:** bioluminescence, hexamerin, keroplatin, reductase

## Abstract

Bioluminescence (BL) in the Keroplatinae subfamily (Diptera: Keroplatidae) is found in *Keroplatus, Neoceroplatus* and *Orfelia fultoni* larvae. In *O. fultoni* , BL involves an oligomeric luciferase, a luciferin called *keroplatin*, which is associated with a substrate binding fraction (SBF), whose molecular identity and function remain uncertain. Non‐luminescent web‐constructing predatory larvae of *Neoditomyia* sp. (Keroplatinae) also contain keroplatin and SBF in their bodies, suggesting additional unknown roles for this compound in this subfamily. To identify gene products differentially expressed between luminescent and non‐luminescent larvae, especially those associated with luciferase, SBF and keroplatin synthesis, here we compared the transcriptional and proteomic profiles of *Neoditomyia* sp., *O. fultoni* and *Arachnocampa* larvae and conducted biochemical assays. Similarly to *O. fultoni* , *Neoditomyia* sp. displays an abundance of hexamerin isoforms and transcripts associated with the tryptophan and kynurenine pathway, which is potentially involved with keroplatin synthesis and silk production. Despite displaying a similar electrophoretic pattern of *Orfelia* luciferase purified fractions, no luciferase activity was detected in *Neoditomyia* purified fractions. The SBF‐enriched fractions from *O. fultoni* and *Neoditomyia* revealed a similar abundance of hexamerins, the presence of flavin‐dependent reductases, keroplatin and riboflavin. The results indicate that the SBF consists of protein aggregates associated with riboflavin and keroplatin, which is used as luciferin in bioluminescent species and for other still unveiled physiological functions in non‐luminescent species.

## Graphical abstract

- Transcriptomic and proteomic comparison of luminescent *O. fultoni* and non‐luminescent *Neoditomyia* sp show that the substrate binding fraction (SBF) has a similar abundance of hexamerins and the presence of flavin‐dependent reductases.
- In addition to the high content of keroplatin, SBF also contains considerable amounts of riboflavin and displays reductase activity.
- The SBF reductases may participate in the reduction or synthesis of keroplatin and other compounds.

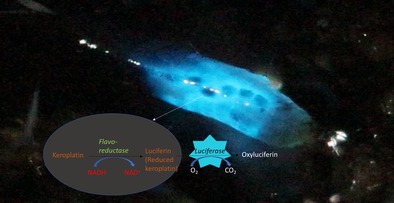

## INTRODUCTION

Bioluminescence in Diptera is found in two subfamilies of the Keroplatidae family: Arachnocampinae (*Arachnocampa*), predominantly found in Oceania, and Keroplatinae (*Keroplatus, Neoceroplatus* and *Orfelia*) (Fulton, 1941; Matile, 1990; Viviani et al., 2002) that are distributed worldwide. Noteworthy, the bioluminescent systems of these subfamilies are both morphologically and biochemically distinct, indicating distinct evolutive origins (Viviani et al., 2002). Both luminescent and non‐luminescent larvae of this subfamily also produce silk to construct webs for prey capture in Keroplatidae larvae (Sutherland et al., 2010).

*Arachnocampa* larvae build webs on cave roofs in New Zealand and Australia, employing their blue‐green bioluminescence to lure prey to their webs (Gatenby, 1959; Viviani et al., 2002). The lanterns of *Arachnocampa* are a modification of the terminal ends of Malpighian tubules (Gatenby, 1959). The bioluminescent system comprises a luciferase, ATP, magnesium (Viviani et al., 2002) and a luciferin, which is a derivative of xanthurenic acid and tyrosine (Watkins et al., 2018). Transcriptomic studies on lanterns from *Arachnocampa* larvae indicated that its luciferase may belong to the superfamily of adenylate forming enzymes, the same family as the luciferase found in Coleoptera (Sharpe et al., 2015; Silva et al., 2015).

Similarly to *Arachnocampa*, the larvae of *Orfelia fultoni* use light to attract small invertebrates to their webs along riverbanks and waterfalls in the Appalachian Mountains (Fulton, 1941). The blue bioluminescence (460 nm) of *O. fultoni* larvae is associated with proteinaceous black bodies, supposedly of mitochondrial origin (Bassot, 1978). Previous studies have shown that the bioluminescence system of *Orfelia* involves a 140 kDa heterodimeric luciferase, a luciferin, and a substrate binding fraction (SBF) which is associated with luciferin (Viviani et al., 2002). The *Orfelia‐*type luciferin was also found in the non‐bioluminescent larvae of the Neotropical *Neoditomyia* sp. (Viviani et al., 2018) and for this reason was named *keroplatin* (Viviani et al., 2020), suggesting that this compound may play additional yet unknown roles in non‐bioluminescent species within the Keroplatinae subfamily (Viviani et al., 2018, 2020).

More recently, purification and native PAGE suggested that the functional luciferase has ~220 kDa, possibly consisting of a trimeric structure composed of ~60 to 78 kDa monomers (Viviani et al., 2020). Proteomic analysis showed that the luciferase fractions are particularly enriched with hexamerins, the same occurring with SBF, which composes the black bodies, indicating the involvement of hexamerins in bioluminescence (Amaral et al., 2021; Viviani et al., 2020).

Hexamerins belong to a superfamily that is believed to have evolved from crustacean haemocyanins, losing their capacity to bind oxygen (Burmester et al., 1998). This superfamily includes arthropod prophenoloxidases and haemocyanins, insect hexamerins and Diptera hexamerin receptors (Burmester, 1999). Some hexamerins can also bind small organic compounds, such as riboflavin and xenobiotics (Burmester et al., 1998; Haunerland & Bowers, 1986).

Similarly to *O. fultoni*, larvae and pupae of Euroasiatic *Keroplatus* species emit a weak blue bioluminescence associated with proteinaceous granules in the fat body (Baccetti et al., 1978). Recently, the first bioluminescent species of Keroplatidae, *Neoceroplatus betaryiensis*, closely related to Euroasiatic *Keroplatus* spp., was discovered in Brazil (Falaschi et al., 2019). These larvae live on branches or decaying tree logs, where they lie between the wood and their secreted mucus, and emit blue bioluminescence (472 nm) from their last abdominal segment and two regions located laterally on the first thoracic segment. The luciferin and luciferase cross‐reaction between *Orfelia* and *Neoceroplatus* cold‐hot extracts, and by inference of the closely related *Keroplatus* spp., revealed that these genera share the same bioluminescent system. Molecular phylogeny suggests that bioluminescence evolved only once in the Keroplatinae subfamily and that *Neoditomyia* represents a basal clade of the Keroplatinae subfamily (Falaschi et al., 2019; Viviani et al., 2018).

Recently, Kotlobay et al. (2023) reported evidence that 3‐hydroxykynurenic acid is the oxyluciferin of the bioluminescent reaction. A branch of this pathway involves kynurenic acid synthesis by indol‐pyruvate intermediates from tryptophan (Zsizsik & Hardeland, 2002).

Furthermore, the same group reported the accumulation of riboflavin in larvae of *Keroplatus testaceus* (Kotlobay et al., 2022), but its function in bioluminescence remains uncertain. Several insect groups accumulate this compound for the homeostasis of FAD and FMN during the feeding period. In *Drosophila melanogaster* (Diptera), riboflavin is stored in granules (riboflavin granules) (Nickla, 1972; Zhang et al., 2018).

Despite the above studies, the molecular identities of the luciferase, SBF and luciferin (*keroplatin*), as well as other potential cofactors involved in the bioluminescence of Keroplatinae subfamily members, still await confirmation. While transcriptional analysis has been performed for the bioluminescent *O. fultoni* (Amaral et al., 2021), a more comprehensive understanding of the molecular requirements of the bioluminescence system of Keroplatinae could be greatly benefited from transcriptional, proteomic and biochemical studies comparing bioluminescent and non‐bioluminescent species of this subfamily, and from the Keroplatidae family as a whole.

Therefore, in this study, we compared the transcriptional and proteomic profiles of the non‐luminescent *Neoditomyia* sp. larvae with those previously published for the closely related bioluminescent *O. fultoni* (Amaral et al., 2021) and the more distantly related bioluminescent *A. luminosa* from the Arachnocampinae subfamily (Sharpe et al., 2015). Additionally, we analysed SBF and luciferase‐enriched fractions for enzymatic activities and compounds potentially associated with the bioluminescence.

## EXPERIMENTAL PROCEDURES

### Insects and total RNA extraction

*Neoditomyia* sp. larvae were collected from the cave roofs at Intervales State Park and PETAR (São Paulo state, Brazil). Total RNA was extracted from the whole body of five larvae using the TRIzol reagent (Life Tech., USA) following the protocol of the manufacturer. The total RNA quantity at 260 nm and quality ratio 260/280 nm were measured using a NanoView spectrophotometer (GeHealth, USA) and further assessed by an Agilent 2100 Bioanalyzer (Agilent Tech., USA).

### cDNA libraries construction and Illumina sequencing

The mRNA isolation and the cDNA library construction were carried out at the NGS Soluções Genômicas facility (Piracicaba, Brazil), utilising the TruSeq Stranded mRNA kit (Illumina, USA). Paired‐end sequencing (2 × 100 bp) of transcriptome libraries was performed on a NextSeq instrument (Illumina, USA), generating an output of 30 million reads per sample. The raw reads were deposited on the NCBI database under the project number PRJNA1039798.

### De novo transcriptome assembly, functional annotation, transcript abundance and computational analysis of sequences

The raw reads quality was verified by FastQC 0.11.5 software (Andrews, 2010), and low‐quality reads (Phred Q ≤ 30) and adaptors were removed using the FASTX‐TOOLKIT 0.0.13 (Pearson et al., 1997). De novo assembly of the filtered reads was performed using the Trinity 2.0.2 software (Grabherr et al., 2011). The transcripts were translated to amino acid by the TransDecoder tool (available at https://github.com/TransDecoder/TransDecoder/releases), and sequences that displayed >95% of similarity were overlapped (unigenes or transcripts) using the software CD‐Hit v4.8.1 (Li et al., 2001).

Subsequently, the transcripts underwent a similarity search against the NCBI non‐redundant (nr) and UNIPROT/SWISS‐PROT databases using the Blastp algorithm in Blast2GO 3.0 software (Conesa et al., 2005). Gene Ontology (GO) terms were assigned with an *e*‐value <10^−6^, annotation cut‐off >55 and graphically represented using the Web Ontology Annotation Plot 2.0 (WEGO) tool (Ye et al., 2018).

Transcript abundance was assessed using FPKM (Fragments Per Kilobase Million) values, calculated with the *align_and_estimate_abundance.pl* script from Trinity. Selected transcripts were aligned using the ClustalW algorithm tool (Thompson et al., 1994) in Mega 6.0 software (Tamura et al., 2013). Phylogenetic analyses were performed using the IQ‐Tree v1.6.12 software (Minh et al., 2020), which also inferred the most appropriate substitution model for each alignment. The resulting phylogenetic tree was visualised using FigTree v.1.3.1 software (Rambaut, 2007).

### PCR analysis of hexamerins sequences

The total RNA extracted from *Neoditomyia* larvae was also utilised to synthetise the corresponding cDNA using the GoScript™ Reverse Transcription System kit (Promega, USA). The cDNA served as template for PCR amplifications of sequences similar to hexamerins (~35%–60% of identity) from the transcriptome (comp7074_c0_seq1, comp8442_c0_seq1, comp10266_c0_seq1, comp10373_c0_seq) using specific primers outlined in Data S1.

PCR reactions were carried out using a PCR Master Mix kit (Promega, USA), with the following conditions: initial denaturation of 5 min at 95°C, 30 cycles of 1 min at 95°C (denaturation), 1 min at 50°C (annealing) and 1 min at 72°C (extension), and a final step of 10 min at 72°C. Amplification results were analysed by 1% (m/w) agarose gel electrophoresis in TAE 1× (20 mM Tris‐acetate and 0.5 mM EDTA) buffer and visualised by fluorescence using a UV transilluminator. The PCR reaction products were further confirmed by Sanger DNA sequencing.

### Transcriptome analysis of bioluminescent Diptera species

A comparative analysis of the new transcriptome of *Neoditomyia* sp. with those previously published from bioluminescent Arachnocampinae and Keroplatinae species was conducted. For this purpose, we re‐examined the transcriptomes of *O. fultoni* (PRJNA578979; Amaral et al., 2021) and *A. luminosa* (PRJNA290397; Sharpe et al., 2015) to search for homologous genes and their abundance values in these species.

### Proteomic analysis of *Neoditomyia* sp.

Partially purified samples from *Neoditomyia* extracts were electrophoresed in 7.5% SDS‐PAGE, and the resulting gel bands between 70 and 75 kDa were excised for subsequent proteomic analysis (Viviani et al., 2020). After electrophoresis, the SDS gel was stained with colloidal blue. The amount of protein of each band was estimated to be between 50 and 500 ng. These bands were carefully excised from the gel, briefly washed with 50% acetonitrile, and stored in microcentrifuge tubes in dry ice. The samples were shipped to Prof. D. Hayes McDonald from the Proteomics laboratory at Vanderbilt University for MS analysis. Proteomic data of *Neoditomyia* were cross‐checked against the RNA‐seq data from *O. fultoni* for protein identification (Amaral et al., 2021). The identification of known protein matches was carried out using Scaffold 5 software (Searle, 2010) and BLASTp.

### Luciferase and SBF purification by anion‐exchange chromatography

To purify luciferase from *Orfelia*, SBF from *Neoditomyia* sp. larvae, and to verify the possible presence of similar luciferase‐like enzymes in the latter, an adaptation of the previously used anion‐exchange chromatography protocol was used (Viviani et al., 2020). Five larvae were homogenised in extraction buffer (0.10 M sodium phosphate, Triton X‐100 1% and 1 mM EDTA) and centrifuged at 15,000 × *g*. After the centrifugation, the *O. fultoni* and *Neoditomyia* sp. crude extracts were ammonium sulphate fractioned, and the fractions precipitated at 50% (SBF‐enriched fraction) and 70% saturation (luciferase‐enriched fraction) were resuspended and dialysed in 25 mM Tris–HCl buffer pH 8.0. The dialysed samples were loaded onto a HiTrap Q HP column (GE Healthcare, USA) equilibrated with buffer A (25 mM Tris–HCl pH 8.0) and a linear gradient of buffer B 0.1–0.3 M NaCl (25 mM Tris–HCl pH 8.0) was applied, using an AKTA start chromatograph (Cytiva, USA). The eluted fractions were used for luminometric assays for luciferase, and electrophoresed in 7.5% SDS‐PAGE. The fraction eluted in the flowthrough was enriched in SBF and that eluted with 0.3 M NaCl contained the luciferase.

### Keroplatin (luciferin) extraction

The hot extract containing luciferin (keroplatin) was prepared by homogenising four *Neoditomyia* sp. larvae in a Potter Elvejem homogeniser containing ice‐cold ultrapure water with 10 mM DTT. The extract was transferred into a glass sealed with a rubber cover and vacuum‐sealed and then incubated at 98°C for 5 min. After that, the hot extract was cooled on ice and centrifuged at 15,000 × *g* at 4°C for 15 min. After the centrifugation, the supernatant (hot extract) was stored under vacuum in a blood collecting‐type vessel at −20°C.

### Luciferase assays

The luciferase activity was determined luminometrically, according to Viviani et al. (2020). The assay was carried out by mixing 5–10 μL of crude extract or purified *Orfelia* luciferase, 2.5 μL of keroplatin and 87.5 μL of Tris–HCl 0.10 M pH 8.0 in a luminometer tube. The luminescent intensity was measured in *cps* (counts per second) using an AB2200 luminometer (Atto, Japan).

### Riboflavin analysis

Riboflavin presence was analysed by fluorescence under UV light (370 nm) irradiation, and by fluorescence spectra using a Hitachi F4500 spectrofluorometer upon excitation at 450 nm and emission spectra scanning between 470 and 600 nm. Furthermore, analysis of the presence of riboflavin in the partially purified samples was performed after Thin‐Layer Chromatography (TLC) on silica gel plates using ethyl acetate, ethanol and water (5:3:2) as a moving phase in the presence of standard riboflavin (Sigma‐Aldrich, USA), followed by fluorescence analysis upon UV light irradiation.

### Reductase assays

Reductase activity was assayed in the presence of 2,6‐dichlorophenolindophenol (DCPI) and reduced nicotinamide adenine dinucleotide (NADH (Sigma‐Aldrich, USA). In the assay, 5 μL of 10 mM DCPI, 5–10 μl of SBF‐enriched fraction or purified luciferase were mixed with 82.5 μL of 0.10 M sodium phosphate buffer pH 7.5 and incubated for 5 min at room temperature. The colour change from blue to colourless of DCPI was taken as an indication of reductase activity. The addition of 2.5 mM NADH further increased the speed of the colour change in the presence of reductase. As a control, the SBF‐enriched or luciferase fractions were replaced by the respective extraction buffers. The assays were repeated 3 times.

### Effect of SBF‐enriched fractions on keroplatin (luciferin) activity

The remaining bioluminescence activity of keroplatin incubated with SBF‐enriched fractions from *Neoditomyia* and *O. fultoni* was imaged in a CCD camera. For this purpose, 5 μl of *Neoditomyia* keroplatin (luciferin) containing hot extracts and 10 μL of SBF‐enriched fractions from *Neoditomyia* or *O. fultoni* were added to 83 μL of 0.10 M Tris–HCl buffer pH 8.0 and incubated at 4°C overnight. Then, 2 μL of purified *Orfelia* luciferase was added and mixed into each well, and bioluminescence was imaged using a CCD camera. For the control, 25 mM Tris–HCl buffer pH 8.0 was used instead of the SBF‐enriched fraction. Densitographic analysis of images was done using ATTO CS Analyser and result outputs were provided in counts per second (cps) (Data S2).

## RESULTS

### Transcriptional analysis and abundance of *Neoditomyia* sp.

**FIGURE 1:**
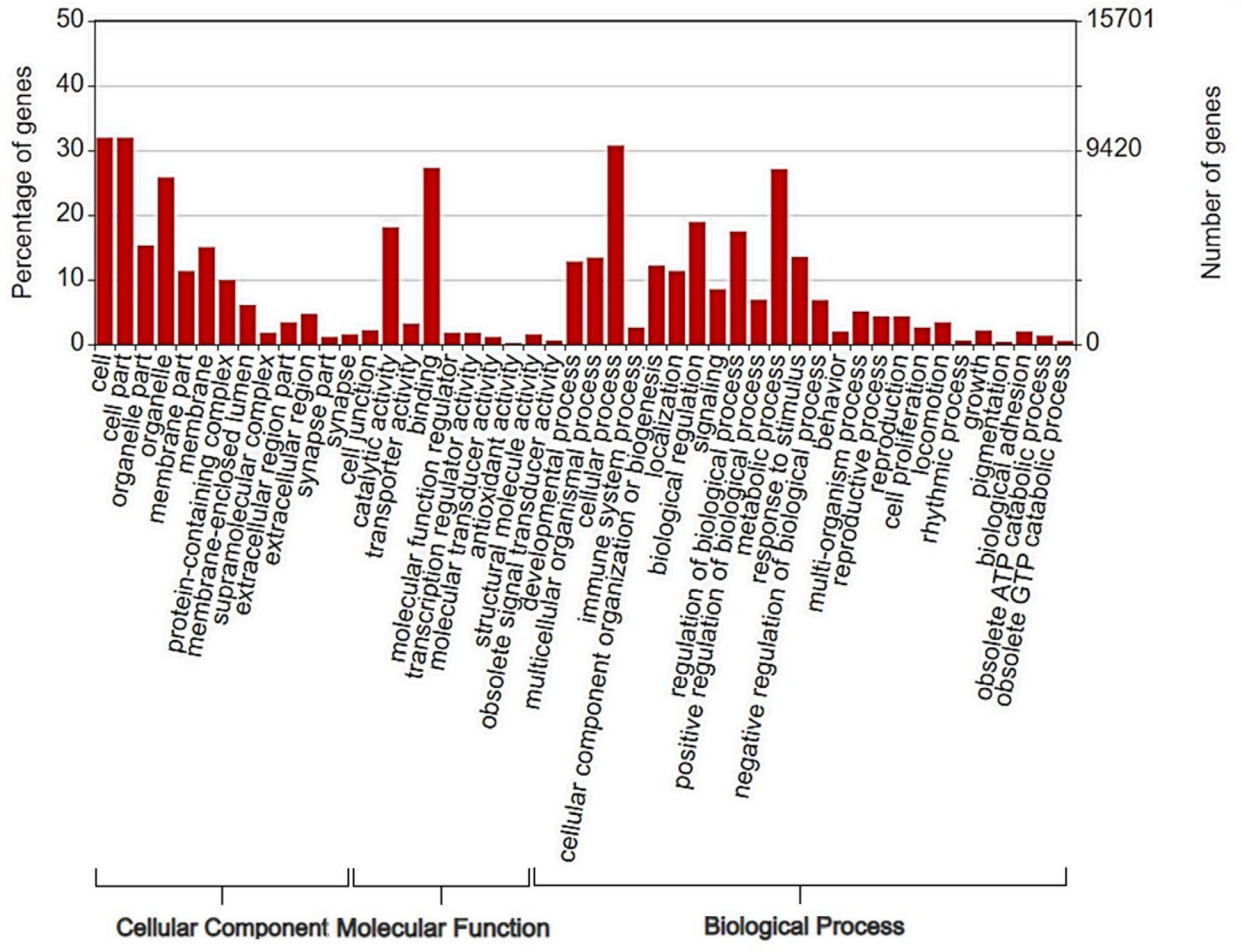
Gene Ontology terms annotation of the non‐redundant transcripts from *Neoditomyia* sp (by WEGO).

The sequencing of the cDNA library from the non‐luminescent cave worm *Neoditomyia* sp. resulted in approx. 62 million raw reads. After data processing and de novo assembly, a total of 31,403 non‐redundant transcripts were obtained. Gene Ontology annotation, covering molecular function, biological process or cellular component from these non‐redundant transcripts, resulted in 12,646 (40.24%) annoted transcripts (Figure 1). Notably, among them, 18,757 transcripts (59.72%) lacked assigned GO terms, indicating a gap of knowledge about the functions of several gene/proteins in these species. The GO terms profile of *Neoditomyia* transcriptome was similar to the profile of *O. fultoni* transcriptome (Amaral et al., 2021), as expected given their close phylogenetic relationship and similar ecological and behavioural characteristics, such as predation, web construction and wet habitats.

The thirty most abundant transcripts in *Neoditomyia* are shown in Table 1. The top‐ranking transcripts are similar to *omega‐amidase NIT2* (FPKM 19,764.18; 8327.27), which are orthologs of the human protein Nitrilase Family Member 2. *Omega‐amidase NIT2* (*NIT2*) enzyme converts α‐ketoglutaramate, a metabolite produced by glutamine transamination, to α‐ketoglutarate by deamidation (Herrle et al., 2024; Krasnikov et al., 2009). This enzyme is related to nitrogen and sulphur metabolism, but its function is not clearly understood in animals (Krasnikov et al., 2009). Recently, it was suggested that the reaction catalysed by human *NIT2* is redox‐sensitive in endothelial cells exposed to H_2_O_2_ (Herrle et al., 2024). The CG8132 gene from *Drosophila* codes for an omega‐amidase that is orthologous to human *NIT2*, and it is related to eye and wing development in this species (Pletcher et al., 2019; Rotelli et al., 2019). *NIT2* was also differentially expressed in the *O. fultoni* day‐time transcriptome (Amaral et al., 2021). In contrast, the *Drosophila* gene (CG8132) displays low abundance and enrichment in the whole body during the larval stage (Krause et al., 2020; Pletcher et al., 2019). It is possible that *NIT2* is related to some ecological adaptation found in Keroplatinae species.

**TABLE 1:** Top 30 most abundant transcripts in *Neoditomyia* sp. larvae.

Contig number	Putative functional annotation	FPKM
comp6168_c0_seq1	Omega‐amidase NIT2	19764.18
comp6696_c0_seq1	Glycerol‐3‐phosphate phosphatase	9812.59
comp10552_c0_seq4	Putative serine protease inhibitor 4	8923.34
comp7939_c0_seq2	Omega‐amidase NIT2	8327.27
comp10291_c0_seq1	Aldehyde dehydrogenase 1	8246.60
comp10291_c0_seq2	Aldehyde dehydrogenase 1	7395.11
comp10570_c0_seq5	Uncharacterised protein	5184.99
comp10120_c0_seq3	Actin	4705.34
comp11111_c0_seq1	Succinate‐semialdehyde dehydrogenase	4444.42
comp6480_c0_seq1	Cytochrome c oxidase subunit 1	4343.40
comp8442_c0_seq1	Hexamerin	4322.25
comp10169_c0_seq1	Peritrophin‐1	4106.16
comp5144_c0_seq1	DM5 domain‐containing protein	3936.71
comp10434_c0_seq3	Translation initiation factor IF‐2	3678.14
comp7103_c0_seq1	Translationally‐controlled tumour protein homologue	3327.19
comp10322_c0_seq1	Serine protease inhibitor (serpin) 10	2838.81
comp10266_c0_seq1	Larval serum protein 2	2658.10
comp6873_c0_seq1	Elongation factor 1‐alpha	2525.73
comp6831_c0_seq1	Myosin light chain 2	2455.84
comp6425_c0_seq1	Cytochrome b	2294.97
comp10899_c0_seq1	40S ribosomal protein S4	2157.70
comp11424_c0_seq1	No similarity found	2061.56
comp5351_c0_seq1	No similarity found	2053.52
comp7595_c1_seq2	Tubulin alpha chain	1939.88
comp10434_c0_seq2	No similarity found	1718.78
comp10759_c0_seq1	40S ribosomal protein SA	1715.74
comp6904_c0_seq1	60S ribosomal protein L4	1709.00
comp9322_c0_seq1	60S ribosomal protein L7a	1637.68
comp10843_c0_seq1	40S ribosomal protein S3a	1604.43
comp10641_c0_seq1	60S ribosomal protein L22	1587.28

Transcripts similar to ribosomal proteins and catabolic oxidoreductases, including *aldehyde dehydrogenase 1*, respiratory chain *3‐succinate‐semialdehyde dehydrogenase, cytochrome c* and *cytochrome b*, predominate among the most abundant transcripts (Table 1).

### Gene products potentially related to bioluminescence

#### Hexamerins

Previously, hexamerins were found in partially purified samples of *Orfelia* luciferase and SBF (Viviani et al., 2020). As expected, similar transcripts to hexamerins/larval serum proteins were also found in the *Neoditomyia* transcriptome. Four transcripts similar to hexamerins were also found in the *Neoditomyia* transcriptome (comp7074_c0_seq1 FPKM: 85.03, comp8442_c0_seq1 FPKM: 4322.22, comp10266_c0_seq1 FPKM: 2658.1, comp10373_c0_seq1 FPKM: 651.37). These protein sequences display distinct identities among them (28–51%) (Table 2). These transcripts from *Neoditomyia* were also amplified by RT‐PCR, confirming their high expression Data S3.

**TABLE 2:** Comparison of transcripts similar to hexamerins from *Neoditomyia* sp. and *O. fultoni* . Identities were calculated from the translated amino acid sequences.

Transcript ID	comp7074	comp8442	comp10266	comp10373	Orf 715	Orf 750
Neod comp7074	/	32%	28%	40%	28%	30%
Neod comp8442	32%	/	51%	34%	52%	81%
Neod comp10266	28%	51%	/	30%	83%	51%
Neod comp10373	40%	34%	30%	/	31%	33%
Orf 715	28%	52%	83%	31%	/	53%
Orf 750	30%	81%	51%	33%	53%	/

The comparison of these transcripts with two main hexamerins isoforms found in the *Orfelia* transcriptome: Orf_715 and Orf_750, coding 715 and 750 amino acids respectively (Amaral et al., 2021) showed a high identity percentage between the transcript comp8442 and Orf_750 (81%); and between the transcript comp10266 and Orf_715 (82%). Such higher identities between the hexamerin sequences from different species rather than from the same species (Table 2) indicate that they could be alloforms of the same protein. Furthermore, the transcripts comp8442 (FPKM: 4322.25) and comp10266 (FPKM: 2658.10) are among the most abundant transcripts in *Neoditomyia* larvae. Other analysed hexamerins from Diptera species (*A. luminosa*, *Culex quinquefasciatus* and *Aedes aegypti*) displayed about 40%–58% identity with *Orfelia* hexamerins, and 42–58% about with *Neoditomyia* hexamerins.

#### Riboflavin‐binding hexamerins

Considering that riboflavin was found to be abundant in the bioluminescent relative, *Keroplatus testaceous* (Kotlobay et al., 2022) and that some hexamerins are involved in binding to riboflavin (Burmester et al., 1998; Haunerland & Bowers, 1986), we compared the *Neoditomiya* and *Orfelia* hexamerin sequences with the riboflavin‐binding hexamerins (RBHs). The hexamerins of *Neoditomyia* sp. (comp10266 and comp8442), *O. fultoni, A. luminosa*, *C. quinquefasciatus* and *A. aegypti* display ~30% identity with the RBH of *Hyalophora cecropia* (Maggee et al., 1994; Rao et al., 2016). The RBH primary structure from *Corcyra* (Lepidoptera) displays three conserved amino acid residues (Asp176, Ile634 and Leu659) that contribute to binding riboflavin (Rao et al., 2016), which are substituted by the conservative Gln180, Val664 and Leu/Tyr698 in *Orfelia* and *Neoditomyia* hexamerins, indicating that these hexamerins could also be involved in riboflavin binding.

#### Structural/functional inferences

**FIGURE 2:**
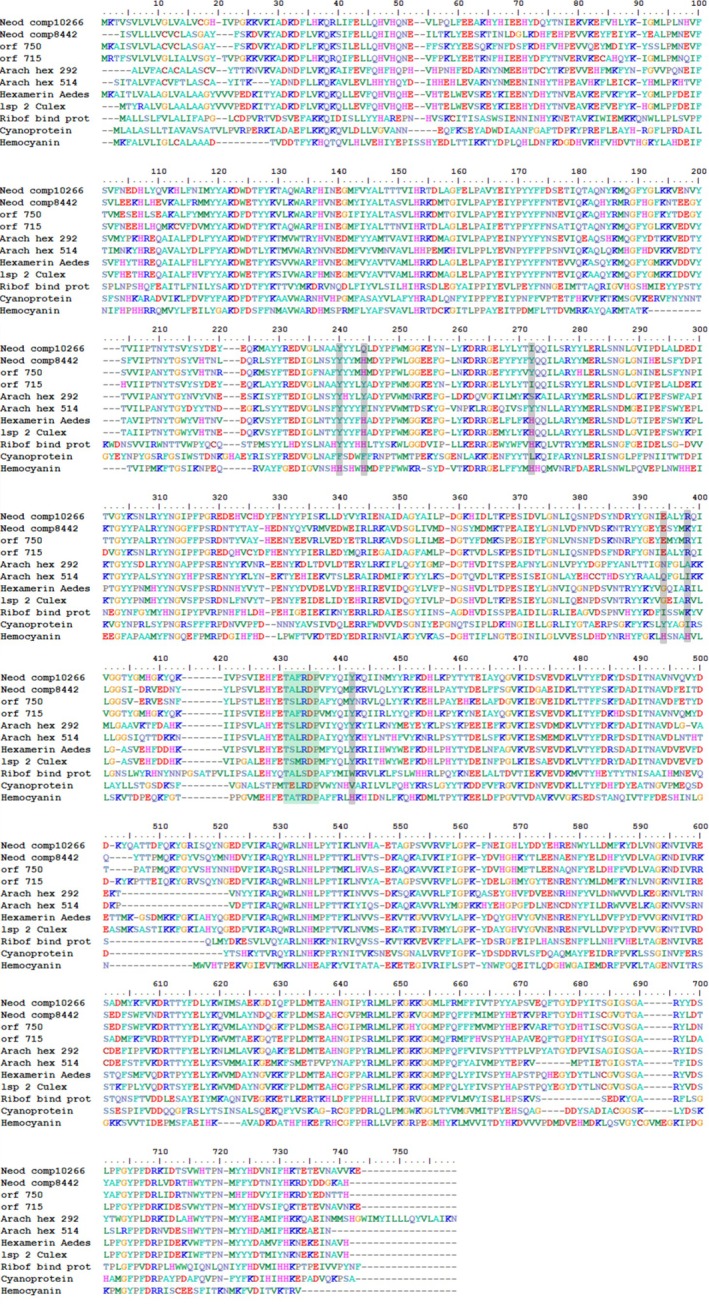
Multialignment of the primary structures of hexamerins from *Neoditomyia sp*, *Orfelia fultoni* (Orf 715 e Orf 750), *A. luminosa* lanterns (Hex 292 e Hex 514) and insect hexamerins from NCBI: *larval serum protein* 2 of *Culex quinquefasciatus* (XP001843494.1), hexamerin of *Aedes aegypti* (XP001661466.1), cyanoprotein of *Riptortus clavatus* (D8727.1), riboflavin‐binding hexamerin of *Hyalophora cecropia* (AAB86645.1) and haemocyanin of *P. marginata* (Q70Q68). In grey: Histidine residues related to binding O_2_ in haemocyanins. In green: Conserved region in insect hexamerins.

The multi‐alignment of transcripts similar to hexamerins of *Neoditomyia* and *Orfelia*, with other insect hexamerins from the NCBI database (Figure 2), and haemocyanin sequence (Q70Q68 *Perla marginata*—Insecta: Plecoptera), showed substitutions in positions corresponding to the six conserved histidine residues responsible for O_2_ binding in haemocyanins (residues in grey in Figure 2) (Pick et al., 2009). As anticipated for insect hexamerins, the lack of such histidine residues in these proteins indicates that they apparently cannot bind oxygen or carry out oxidation reactions. However, some hexamerins exhibit one or two copper‐binding histidines, and the positions of these residues are variable among the protein sequences (Burmester et al., 1998; Pick & Burmester, 2009; Terwiliger et al., 1999).

The transcript comp8442 of *Neoditomya* and transcript Orf_750 of *Orfelia* display just one conserved copper‐binding histidine residue related to oxygen binding (second histidine residue—H244) (Figure 2). On the other hand, the riboflavin‐binding protein from *H. cecropia* (Lepidoptera: Saturniidae) and hexamerins from *A. aegypti* (Diptera: Culicidae) and *C. quinquefasciatus* (Diptera: Culicidae) display two conserved copper‐binding residues (second and third histidine residues—H244 and H272). Among the hexamerins sequences, the most notable conserved region is the motif 431TXX(R)DP436 (Figure 2) in the N‐terminal portion, a motif important for the overall protein structure (Burmester et al., 1998), but whose specific function remains unknown.

**FIGURE 3:**
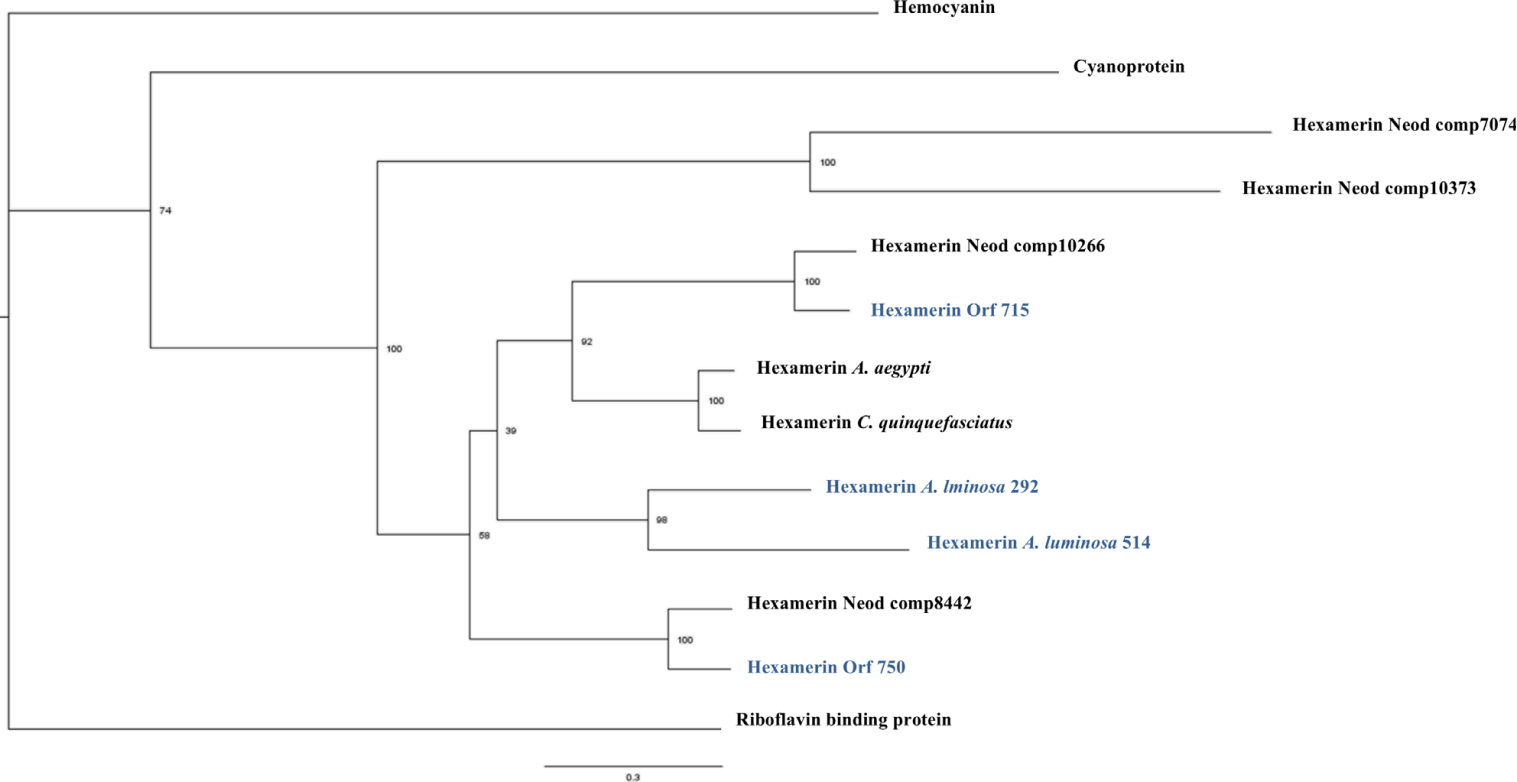
Phylogenetic tree by maximum likelihood of hexamerins. Protein sequences used in the analysis: transcripts similar to hexamerins from the *Neoditomyia* transcriptome (comp7074, comp8442, comp10266, comp10373), transcripts similar to hexamerins from the *Orfelia* transcriptome (Orf 715, Orf 750), haemocyanin of *P. marginata* (Q70Q68); cyanoprotein of *R. clavatus* (D87272.1); hexamerin of *Aedes aegypti* (XP 0001661466.1); larval serum protein 2 of *C. quinquefascitaus* (XP 001849286.1); hexamerins of the clones 292 and 514 from *A. luminosa* lanterns (Silva et al., 2015), riboflavin‐binding protein of *Hyalophora cecropia* (AAB86645.1). In blue: Diptera bioluminescent species.

Phylogenetic relations among the transcripts similar to hexamerins of *Neoditomyia* and *O. fultoni*, along with other insect hexamerins, were inferred using protein sequences classified in the hexamerins superfamily (Figure 3). Hexamerins of *Neoditomyia* and *Orfelia* displayed three different strongly supported groups according to the identities among these sequences, indicating that hexamerin genes were duplicated in different evolutionary stages and evolved independently. Proteins from other Diptera species, such as the luminescent relative *A. luminosa* (Keroplatidae: Arachnocampinae) and the non‐luminescent distant relatives *A. aegypti* and *C. quinquefasciatus* (Diptera: Culicidae), also show high support values. The relationship between riboflavin‐binding protein and other hexamerins is difficult to infer. This challenge was also reported by Burmester et al. (1998), as RBHs seem to be closer to the haemocyanins.

### Gene products related to kynurenine metabolism

Considering that the putative oxyluciferin of *Keroplatus* is 3‐hydroxykynurenic acid (Kotlobay et al., 2023), and kynurenine derivatives are produced from tryptophan metabolism through enzymatic and non‐enzymatic reactions, we searched for gene products in this pathway potentially related to the synthesis and metabolism of luciferin (keroplatin) in these organisms (Han et al., 2007; Zsizsik & Hardeland, 2002).

**FIGURE 4:**
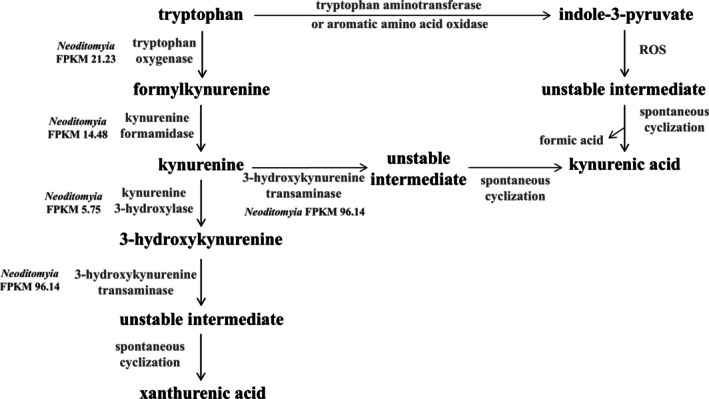
Tryptophan metabolism and the formation of kynurenic and xanthurenic acids. The transcripts similar to the enzymes of this pathway in the *Neoditomyia* transcriptome and their abundance values are also shown. ROS: reactive oxygen species. Adapted from Zsizsik and Hardeland (2002).

As expected, enzymes *tryptophan oxygenase* (comp6926_c0_seq1 FPKM 21.23), *kynurenine formamidase* (comp8900_c0_seq1 FPKM 14.48), *3‐hydroxykynurenine transaminase (3‐HKT)* (comp6117_c0_seq1 FPKM 66.07, comp8263_c0_seq2 FPKM 96.14) and *kynurenine 3‐hydroxylase* (comp18640_c0_seq1 FPKM 5.75) were found in the *Neoditomyia* transcriptome (Figure 4). The most abundant enzyme of this pathway in *Neoditomyia* is the *3‐HKT* (comp8263_c0_seq2 FPKM 96.14), which can either convert kynurenine to kynurenic acid or 3‐hydroxykynurenine to xanthurenic acid (Zsizsik & Hardeland, 2002). Similarly to the *Neoditomyia* transcriptome, the *Orfelia* transcriptome also showed such enzymes. However, these transcripts were not differentially expressed or abundant in the *Orfelia* transcriptome (Amaral et al., 2021).

### Proteomic and biochemical comparison of *Neoditomyia* and *Orfelia*

To identify potential candidates involved in bioluminescence, we also compared the proteomic profiles of *Neoditomyia* SBF‐enriched fractions with the partially purified fractions of SBF and luciferase from *O. fultoni*, previously published (Viviani et al., 2020). Extracts from *Neoditomyia* were partially purified by using ammonium sulphate and anion‐exchange chromatography to perform proteomic analysis and biochemical assays.

#### Luciferase

**FIGURE 5:**
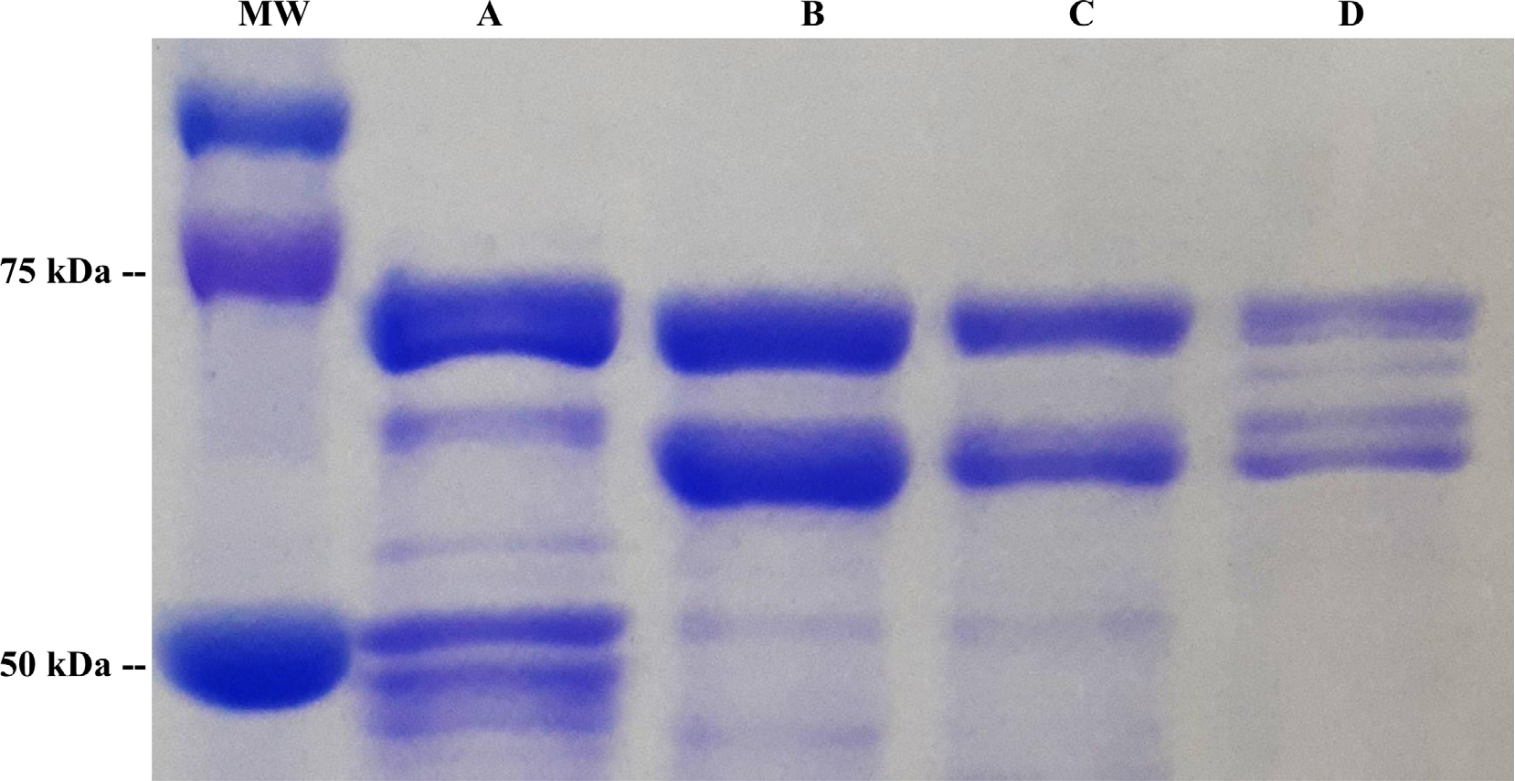
SDS‐PAGE of the purified fractions of *Neoditomyia* sp. crude extract and luciferase fraction of *Orfelia fultoni* . MW: molecular weight; (a) *Neoditomyia* outflow from anion‐exchange chromatography (SBF); (b) purified fraction 1 of *Neoditomyia*; (c) purified fraction 2 of *Neoditomyia*; (d) luciferase‐enriched fraction of *O. fultoni* that was purified by anion‐exchange displaying five protein bands.

In order to look for potential luciferase‐like enzymes in *Neoditomiya* sp, we purified both *Orfelia* luciferase and *Neoditomiya* crude extracts by anion‐exchange chromatography using an adaptation of a previously published method (Viviani et al., 2020), with an automated protein chromatographer. According to SDS‐PAGE, the anion‐exchange purified *Orfelia* luciferase displayed five protein bands with MW between ~61 and 72 kDa (Figure 5d), as previous results showed. The luciferase fraction displayed a specific luminescence activity of ~5 × 10^11^ *cps/*mg determined luminometrically in the presence of *Neoditomiya* larvae keroplatin‐containing hot extracts.

On the other hand, although the purified fractions from *Neoditomyia* extracts (Figure 5b,c) displayed similar elution and electrophoretic bands of *Orfelia* luciferase (Figure 5d), they did not display luminescence activity in the presence of keroplatin containing hot extracts. The results indicate that *Neoditomyia* sp. may have similar monomeric protein components of *Orfelia* luciferase, but without luminescent activity.

### SBF proteomic composition and biochemical properties

#### Proteomic analysis

The mass‐spectrometry analysis of the SDS‐PAGE isolated protein bands from the SBF‐enriched fractions of *Neoditomyia* showed similar proteomic composition of the previously partially purified SBF and luciferase bands of *O. fultoni* larvae (Amaral et al., 2021; Viviani et al., 2020). The most common protein hits were hexamerins, *heat shock protein 70* and *myosin heavy chain* (Table 3). Both transcriptomes and proteomes of *Neoditomyia* and *Orfelia* SBF also showed the presence of *flavin‐dependent reductases*, including *glucose dehydrogenases* and *cytochrome P450 reductase*. In *Orfelia*, both the transcriptome and proteomic analyses indicated the presence of *cytochrome P450 reductase* (comp53946_c0_seq20) and *glucose dehydrogenases* (comp55510_c1_seq1, comp51882_c0_seq6, comp55085_c0_seq1). The transcriptome of *Neoditomyia* also displayed two transcripts similar to *cytochrome P450 reductase*, and nine transcripts similar to *glucose dehydrogenase* (Table 4), with varying values of abundance.

**TABLE 3:** The protein identification of partially purified bands from *Neoditomyia* sp. (column Band/samples) was performed by cross‐checking against the RNA‐seq data from *O. fultoni* (Amaral et al., 2021).

Band/ samples	Transcripts identified	Number of proteins with identification >95%	Transcript RNA‐seq data from *O. fultoni*	Associated gene product
6	29	17	comp42255 c0 seq1	Larval serum protein
			comp36417 c0 seq1	Hexamerin 1
			comp47009 co seq1	Heat shock protein 70
			comp51169 c1 seq1	Heat shock protein 70
			comp55908 c2 seq1	Apolipophorin
7	28	19	comp42255 c0 seq1	Larval serum protein
			comp36417 c0 seq1	Hexamerin 1
			comp47009 co seq1	Heat shock protein 70
			comp51169 c1 seq1	Heat shock protein 70
			comp43540 c0 seq5	Myosin heavy chain
8	35	18	comp42255 c0 seq1	Larval serum protein
			comp36417 c0 seq1	Hexamerin 1
			comp47009 co seq1	Heat shock protein 70
			comp51169 c1 seq1	Heat shock protein 70
			comp43540 c0 seq5	Myosin heavy chain
9	37	13	comp42255 c0 seq1	Larval serum protein
			comp36417 c0 seq1	Hexamerin 1
			comp47009 co seq1	heat shock protein 70
			comp51169 c1 seq1	Heat shock protein 70
			comp43540 c0 seq5	Myosin heavy chain

**TABLE 4:** Transcripts similar to cytochrome P450 reductases and glucose dehydrogenases from the *Neoditomya* sp. transcriptome and their abundance values.

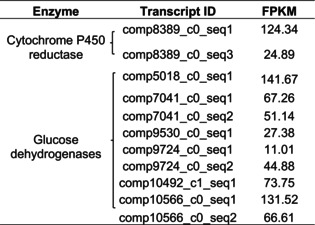

#### Luciferin content and preservation activity

**FIGURE 6:**
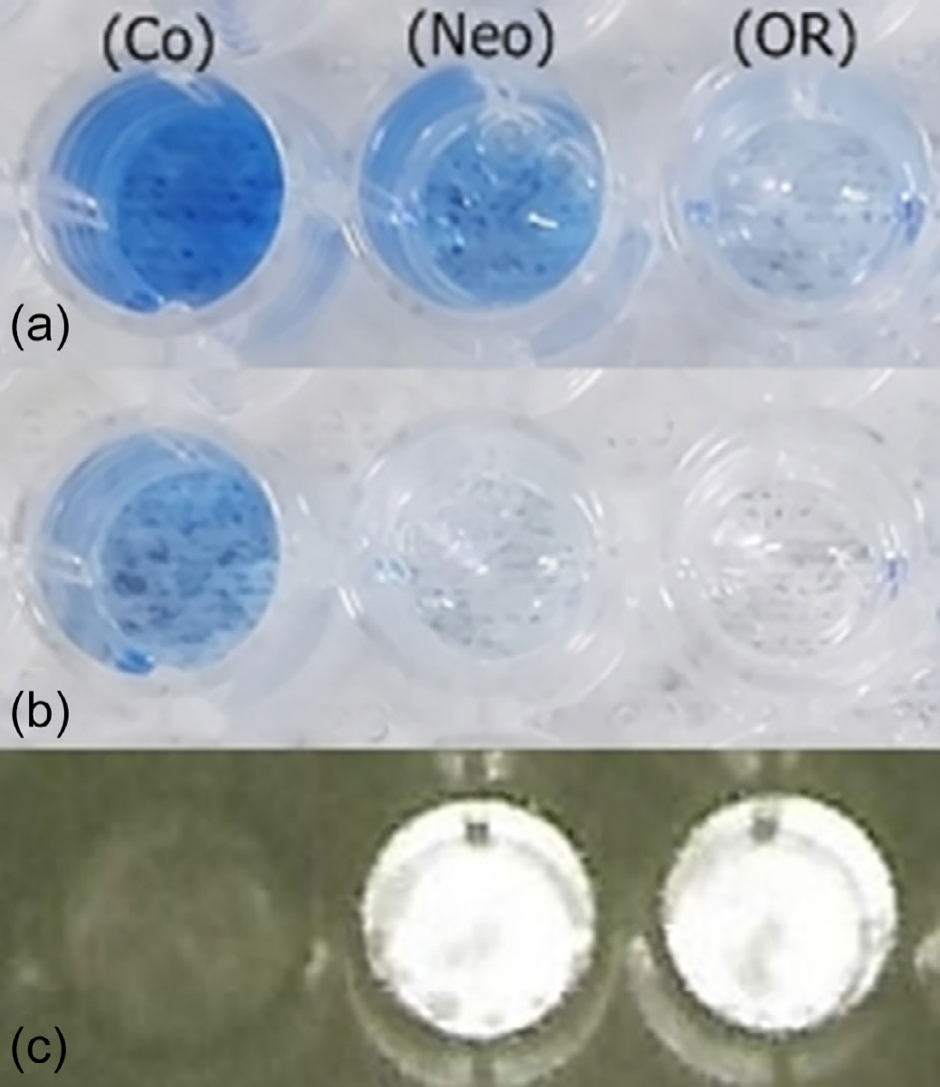
Reductase activity of SBF‐enriched fractions of *Neoditomyia* and *Orfelia fultoni* : (A and B) revealed by colorimetric reduction of DCIP; (a) time 0; (b) after 10 min; (c) CCD camera image of remaining bioluminescence activity of keroplatin (luciferin) incubated with SBF‐enriched fractions overnight at 4°C, upon mixing with *O. fultoni* purified luciferase; (Co) control; (Neo) *Neoditomyia* anion‐exchange chromatography eluted fraction; (OR) *O. fultoni* anion‐exchange chromatography eluted fraction.

Besides containing keroplatin (luciferin), we found that SBF‐enriched fractions, when mixed with additional keroplatin‐containing hot extracts, preserve its luciferin activity, as seen by the luminescent activity displayed upon mixing it with purified *Orfelia* luciferase. In control experiments, in which extraction buffer was used instead of SBF, the luciferin activity was quickly lost due to oxidation (Figure 6; Data S2).

#### Riboflavin content

Considering the recent finding of the abundance of riboflavin in *Keroplatus testaceous* larvae (Kotlobay et al., 2022), here we searched for the presence of riboflavin in *Neoditomyia* and *Orfelia* partially purified fractions. Previously, anion‐exchange chromatography of *Neoditomyia* and *Orfelia* larvae hot extracts showed that, whereas keroplatin (luciferin) eluted at very high salt concentration, the flow‐through displayed yellow‐green fluorescence (Viviani et al., 2020), indicating the presence of riboflavin.

The *Orfelia* purified luciferase fractions did not display any fluorescence indicative of the presence of riboflavin. On the other hand, we found that riboflavin is associated with the SBF. During the partial purification of both *O. fultoni* and *Neoditomyia* larval crude extracts, the 40% ammonium sulphate SBF‐enriched fractions, which consist of a jelly‐like interface containing proteins and lipids, displayed intense yellow‐green fluorescence upon UV light irradiation. Fluorescence spectra and TLC confirmed the presence of riboflavin in these fractions (*λ*_Ex_ = 450 nm; *λ*_FL_ = 525 nm).

#### Flavin‐dependent reductases

Consistent with the proteomic analysis and the presence of riboflavin, the SBF‐enriched fractions displayed flavin‐dependent reductase activity. Visual analysis using the electron‐acceptor dye, DCPI, showed that the freshly prepared SBF fractions of both *Neoditomyia* and *Orfelia* displayed reductase activity (Figure 6). Such reductase activity was lost upon long‐term incubation of the fractions on ice or at 4°C. Furthermore, the addition of NADH further increased the activity for all the SBF fractions, as observed by the faster colour change.

## DISCUSSION

The transcriptional and proteomic comparison of non‐luminescent *Neoditomyia* sp. and bioluminescent *O. fultoni* larvae may bring valuable insights about the differentially expressed gene products; and therefore about the molecular requirements and origin of bioluminescence in the Keroplatinae subfamily. In this work, we focused on expressed genes that could be related to luciferase, luciferin metabolism and the SBF molecular composition.

As expected, the *Neoditomya* transcriptome also showed an abundance of transcripts similar to hexamerins, also called larval serum proteins. Two main hexamerin isoforms (comp10266 and comp8442) from the *Neoditomyia* transcriptome showed high identity with the main *Orfelia* hexamerins (ORF 715 and ORF 750), which were previously associated with luciferase. In the *O. fultoni* transcriptome, these hexamerins were highly abundant during night time, when bioluminescence is elicited. *Orfelia* proteomic analysis showed that partially purified luciferase and SBF samples are enriched in these hexamerins (ORF 715 and ORF 750), suggesting their participation in bioluminescence, either as a subunit in the luciferase quaternary structure or in the SBF complexes participating in binding small compounds associated with bioluminescence (Amaral et al., 2021; Viviani et al., 2020). Although the respective *Neoditomiya* hexamerins were also abundant in the proteomic analysis, no luciferase activity was detected in either crude extract or partially purified fractions of these larvae. If one of these hexamerins indeed participates as a catalytic subunit of the *Orfelia* luciferase, the respective substitutions found in the *Neoditomiya* alloform would be, in principle, enough to prevent the luciferase activity.

The presence of transcripts related to the kynurenic acid pathway and tryptophan metabolism in *Neoditomyia* also deserves attention, as they could be related to keroplatin synthesis. Our transcriptomic analysis also showed the abundance of transcripts related to tryptophan metabolism and the kynurenic acid pathway in *Neoditomyia*. Whereas the presence of enzymes of such pathways is expected in animals, the abundance of some enzymes of this pathway could provide some clues about the keroplatin synthesis and metabolism in these species.

In the vegetarian *Drosophila* larvae, the abundance and enrichment of the genes that encode enzymes of this pathway are low, and the gene CG1555 that encodes the enzyme kynurenine 3‐monooxigenase is absent (Krause et al., 2020). On the other hand, in mosquitoes such as *Anopheles gambiae* (Diptera: Culicidae), which have protein‐rich diets, the enzyme *3‐HKT* is highly expressed in the larval stage. This enzyme metabolises the 3‐hydroxykurenine excess from the tryptophan diet, converting it to xanthurenic acid or kynurenic acid (Han & Li, 2002; Zsizsik & Hardeland, 2002), potentially protecting insect tissues from the 3‐hydroxykurenin‐triggered oxidative stress response (Rossi et al., 2005). Similarly, in *Neoditomyia* larvae, which are predators with a protein‐rich diet, the most abundant enzyme of this pathway was *3‐*HKT.

### Composition and biochemical function of SBF

Our results showed that the SBF‐enriched fractions of both *Orfelia* and *Neoditomiya* larvae contain hexamerins, keroplatin, riboflavin and flavin reductases. Because some hexamerins may also act as riboflavin‐binding proteins (Burmester et al., 1998; Haunerland & Bowers, 1986), we do not exclude the possibility that these proteins in the SBF store riboflavin.

Several flavin‐dependent reductases are membrane‐associated, which is consistent with the association of the reductase activity with the SBF‐enriched ammonium sulphate fractions found in the lipid interface. Furthermore, the presence of protein bands of ~64–72 kDa (Figure 5a), in the SDS‐PAGE of SBF‐enriched fractions from *Neoditomyia,* is consistent with the expected MW range of *cytochrome P450‐reductase* (~75 kDa) and *glucose dehydrogenases* (~68 kDa), which were identified in the proteomic/transcriptomic analysis.

The presence of flavin‐dependent reductases in the SBF, as well as their riboflavin cofactors, could be involved in the reduction/recycling of keroplatin, explaining the preservation effect of SBF on keroplatin luminescence activity (luciferin) reported here (Figure 6); and the previously reported luminescence increasing activity effect upon the addition of SBF to the ongoing *Orfelia* luciferin‐luciferase reaction by Viviani et al. (2002).

In *Orfelia*, the reduced keroplatin is used mainly as luciferin for the bioluminescence reaction, whereas in the non‐luminescent *Neoditomyia* larvae, keroplatin may exert another still unknown biological role. Similarly to *O. fultoni* larvae (Bassot, 1978), *Keroplatus* spp. and *Neoceroplatus betaryensis* larvae have developed dark granules (black bodies), which are likely associated with bioluminescence (Falaschi et al., 2019; Osawa et al., 2014). These granules were also observed in non‐luminescent species of *Keroplatus* (Baccetti et al., 1978). In *Orfelia*, these granules were found to be associated with mitochondria (Bassot, 1978; Viviani et al., 2020), and thus, it is not surprising that they contain reductases and a high content of riboflavin derivatives for respiration purposes. On the other hand, whereas *Neoditomyia* larvae do not have such evident black bodies, they display dark pigmented cells along the dorsal part of the body, which could also contain the SBF (Viviani et al., 2020).

Altogether, these transcriptional, proteomic and biochemical analyses shed light on the composition and function of the enigmatic SBF. The SBF, which is the main component of the black bodies in *O. fultoni*, is also found in *Neoditomiya* sp., and consists of protein complexes made of hexamerins, flavin‐dependent reductases and perhaps other proteins, associated with riboflavin and keroplatin. In bioluminescent species like *Orfelia, Keroplatus* and *Neoceroplatus*, the SBF function is related to storing and keeping riboflavin and keroplatin in their reduced form to act as the luciferin in the bioluminescence reaction. In non‐bioluminescent species and also in luminescent ones, the SBF could be involved in the reduction of keroplatin and other compounds for still unknown biological functions in these larvae.

## CONCLUDING REMARKS

The transcriptional profiles of the non‐luminescent *Neoditomyia* sp. and the closely related bioluminescent *O. fultoni* larvae are similar, showing the presence of gene products related to silk‐web synthesis (Data S4), which are important for their predatory habits; abundance of hexamerins, which are likely involved in bioluminescence as well as for other important biological functions; and enzymes related to kynurenic acid metabolism that could be involved in the biosynthesis of keroplatin. Proteomic and biochemical analysis of the SBF‐enriched fractions of both *Orfelia* and *Neoditomyia* sp. indicate that SBF consists of protein complexes composed of hexamerins, flavin‐reductases associated with keroplatin and riboflavin, whose biological function could be the storage of riboflavin and reduced keroplatin to be used as luciferin in the bioluminescent species, and for other still unknown biological purposes in both luminescent and also in non‐luminescent Keroplatinae species.

## AUTHOR CONTRIBUTIONS

**Jaqueline R. Silva:** Conceptualization; methodology; validation; investigation; writing – original draft; visualization. **Gabriel F. Pelentir:** Methodology; validation; investigation. **Danilo T. Amaral:** Methodology; software; formal analysis; writing – review and editing. **Cassius Stevani:** Conceptualization; writing – review and editing. **Vadim R. Viviani:** Conceptualization; methodology; validation; investigation; visualization; writing – original draft; supervision.

## CONFLICT OF INTEREST STATEMENT

The authors declare no conflicts of interest.

## Supporting information


**Data S1.** Designed primers from Neoditomyia transcriptome sequences that are similar to hexamerins: (F) forward primer and (R) reverse primer.

**Data S2.** Luciferin preservation activity by Neoditomiya and Orfelia Anion exchange SBF fractions.

**Data S3.** Agarose gel electrophoresis of the amplification reactions of the transcripts similar to hexamerins from Neoditomyia. (MW) molecular weight standards; (A) transcript comp7074; (B) repeated amplification of the same reaction A, however, with different concentration of magnesium; (C) transcript comp8442; (D) overflowed sample (E) transcript comp10266; (F) transcript comp10373.

**Data S4.** Gene products related to silk production.

## Data Availability

The raw RNA‐seq reads are available in the NCBI SRA (accession number: PRJNA1039798, PRJNA578979, PRJNA290397). https://www.ncbi.nlm.nih.gov/sra/PRJNA1039798, https://www.ncbi.nlm.nih.gov/bioproject/PRJNA578979, https://www.ncbi.nlm.nih.gov/bioproject/?term=PRJNA290397.
