# Supplementary material for: Transcriptomic, proteomic and biochemical comparison of luminescent and non‐luminescent Keroplatinae larvae (Diptera: Keroplatidae)
Source: Insect Mol Biol. 2025 Aug 21;35(1):34–47. doi: 10.1111/imb.70008 (PMC12779207; doi:10.1111/imb.70008)
Supplement: Supplementary file 1 — Data S1. Designed primers from Neoditomyia transcriptome sequences that are similar to hexamerins: (F) forward primer and (R) reverse primer. [file IMB-35-34-s002.doc]

**Transcriptomic, proteomic and biochemical comparison of luminescent and non-luminescent Keroplatinae larvae (Diptera: Keroplatidae)**

Silva, J. R.^a^, Pelentir, G. F.^b^, Amaral, D. T.^c^, Stevani, C.^d^, Viviani, V. R.^*a,b^

^a^Departamento de Física, Química e Matemática, Universidade Federal de São Carlos, Sorocaba, Brazil.

^b^Programa de Pós-Graduação em Biotecnologia, Universidade Federal de São Carlos, Sorocaba, São Carlos, Brazil.

^c^Laboratório de bioinformática para bioprospecção e mineração de dados ômicos, Centro de Ciências Naturais e Humanas, Universidade Federal do ABC (UFABC), Santo André, São Paulo, Brazil.

^d^Departamento de Química Fundamental, Instituto de Química, Universidade de São Paulo, São Paulo, Brazil

**Supplementary data 1:**

**Table SD1:** Designed primers from *Neoditomyia* transcriptome sequences that are similar to hexamerins: (F) forward primer and (R) reverse primer.

| **Transcript ID** | **Primer sequence (F - R)** |
| --- | --- |
| comp7074_c0_seq1 | **F** ATTGTAACGATTATTGTTGTG |
|  | **R** ACATGTTAGGCGTATGCCA |
|  |  |
| comp8442_c0_seq1 | **F** ATCAGTGTTCTCTTGCTTGTTT |
|  | **R** TTTACCATCATCGTAGTCTC |
|  |  |
| comp10266_c0_seq1 | **F** ATGAAGACCGTCTCAGTGC |
|  | **R** TCCTTAACAACAGCATTGACTT |
|  |  |
| comp10373_c0_seq1 | **F** GATTAGTATTGTTATCCATAAC |
|  | **R** TCAGTGTATCGTAGTAATAC |
