# Supplementary material for: Transcriptomic, proteomic and biochemical comparison of luminescent and non‐luminescent Keroplatinae larvae (Diptera: Keroplatidae)
Source: Insect Mol Biol. 2025 Aug 21;35(1):34–47. doi: 10.1111/imb.70008 (PMC12779207; doi:10.1111/imb.70008)
Supplement: Supplementary file 2 — Data S2. Luciferin preservation activity by Neoditomiya and Orfelia Anion exchange SBF fractions. [file IMB-35-34-s003.docx]

**Transcriptomic, proteomic and biochemical comparison of luminescent and non-luminescent Keroplatinae larvae (Diptera: Keroplatidae)**

Silva, J. R.^a^, Pelentir, G. F.^b^, Amaral, D. T.^c^, Stevani, C.^d^, Viviani, V. R.^*a,b^

^a^Departamento de Física, Química e Matemática, Universidade Federal de São Carlos, Sorocaba, Brazil.

^b^Programa de Pós-Graduação em Biotecnologia, Universidade Federal de São Carlos, Sorocaba, São Carlos, Brazil.

^c^Laboratório de bioinformática para bioprospecção e mineração de dados ômicos, Centro de Ciências Naturais e Humanas, Universidade Federal do ABC (UFABC), Santo André, São Paulo, Brazil.

^d^Departamento de Química Fundamental, Instituto de Química, Universidade de São Paulo, São Paulo, Brazil.

^*^Corresponding author: viviani@ufscar.br

**Luciferin preservation activity by *Neoditomiya* and *Orfelia* Anion exchange SBF fractions**


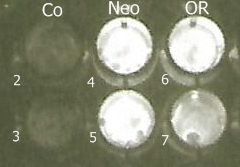


**Densitography analysis**

|  | **Assay** | **Activity (cps)** |
| --- | --- | --- |
| No.1 | Background | 5364 |
| No.2 | Control (Extraction Buffer) | 21494 |
| No.3 | Control (Extraction Buffer) | 19681 |
| No.4 | Anion Exchange fraction 3 | 162686 |
| No.5 | Anion Exchange fraction 3 | 160875 |
| No.6 | Anion Exchange fraction 3 | 163073 |
| No.7 | Anion Exchange fraction 3 | 114876 |
