# Supplementary material for: Transcriptomic, proteomic and biochemical comparison of luminescent and non‐luminescent Keroplatinae larvae (Diptera: Keroplatidae)
Source: Insect Mol Biol. 2025 Aug 21;35(1):34–47. doi: 10.1111/imb.70008 (PMC12779207; doi:10.1111/imb.70008)
Supplement: Supplementary file 3 — Data S3. Agarose gel electrophoresis of the amplification reactions of the transcripts similar to hexamerins from Neoditomyia. (MW) molecular weight standards; (A) transcript comp7074; (B) repeated amplification of the same reaction A, however, with different concentration of magnesium; (C) transcript comp8442; (D) overflowed sample (E) transcript comp10266; (F) transcript comp10373. [file IMB-35-34-s001.docx]

**Transcriptomic, proteomic and biochemical comparison of luminescent and non-luminescent Keroplatinae larvae (Diptera: Keroplatidae)**

Silva, J. R.^a^, Pelentir, G. F.^b^, Amaral, D. T.^c^, Stevani, C.^d^, Viviani, V. R.^*a,b^

^a^Departamento de Física, Química e Matemática, Universidade Federal de São Carlos, Sorocaba, Brazil.

^b^Programa de Pós-Graduação em Biotecnologia, Universidade Federal de São Carlos, Sorocaba, São Carlos, Brazil.

^c^Laboratório de bioinformática para bioprospecção e mineração de dados ômicos, Centro de Ciências Naturais e Humanas, Universidade Federal do ABC (UFABC), Santo André, São Paulo, Brazil.

^d^Departamento de Química Fundamental, Instituto de Química, Universidade de São Paulo, São Paulo, Brazil

^*^Corresponding author: viviani@ufscar.br

**
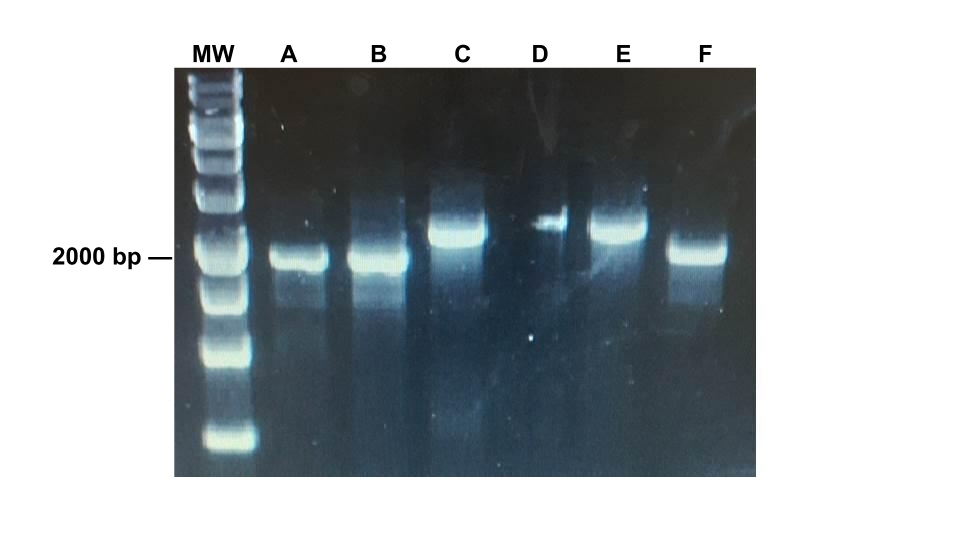
**

**Supplementary data 3** Agarose gel electrophoresis of the amplification reactions of the transcripts similar to hexamerins from *Neoditomyia*. (MW) molecular weight standards; (A) transcript comp7074; (B) repeated amplification of the same reaction A, however, with different concentration of magnesium; (C) transcript comp8442; (D) overflowed sample (E) transcript comp10266; (F) transcript comp10373.
