# Supplementary material for: Transcriptomic, proteomic and biochemical comparison of luminescent and non‐luminescent Keroplatinae larvae (Diptera: Keroplatidae)
Source: Insect Mol Biol. 2025 Aug 21;35(1):34–47. doi: 10.1111/imb.70008 (PMC12779207; doi:10.1111/imb.70008)
Supplement: Supplementary file 4 — Data S4. Gene products related to silk production. [file IMB-35-34-s004.docx]

**Transcriptomic, proteomic and biochemical comparison of luminescent and non-luminescent Keroplatinae larvae (Diptera: Keroplatidae)**

Silva, J. R.^a^, Pelentir, G. F.^b^, Amaral, D. T.^c^, Stevani, C.^d^, Viviani, V. R.^*a,b^

^a^Departamento de Física, Química e Matemática, Universidade Federal de São Carlos, Sorocaba, Brazil.

^b^Programa de Pós-Graduação em Biotecnologia, Universidade Federal de São Carlos, Sorocaba, São Carlos, Brazil.

^c^Laboratório de bioinformática para bioprospecção e mineração de dados ômicos, Centro de Ciências Naturais e Humanas, Universidade Federal do ABC (UFABC), Santo André, São Paulo, Brazil.

^d^Departamento de Química Fundamental, Instituto de Química, Universidade de São Paulo, São Paulo, Brazil.

^*^Corresponding author: viviani@ufscar.br

**Gene products related to silk production**

Whereas the main goal of this work is related to finding gene products associated with the origin of bioluminescence, silk synthesis and web construction is a common important biological trait in predaceous Keroplatidae larvae, which is functionally associated with bioluminescence (prey attraction). Therefore, we also analyzed gene products associated with silk synthesis to find out whether there could be a possible link with bioluminescence. Previously, the Orfelia transcriptomic analyses identified transcripts related to silk formation, such as fibroin heavy chain plus mucin (Amaral et al., 2021). In Arachnocampa, the silk-associated proteins involve collagen/fibronectin plus sialomucin (Amaral et al., 2021). However, although in the Neoditomyia, there were no transcripts similar to fibroin heavy chain and fibroin light chain, we found fibronectin III (comp8162_c0_seq1 FPKM 5.41, comp12742_c0_seq1 FPKM 11.59), salivary secreted mucin (comp9325_c0_seq1 FPKM 932.18), and collagen alpha 1 (comp4933_c0_seq1 FPKM 5.41).

Despite Neoditomyia being phylogenetically closer to Orfelia than to Arachnocampa (Viviani et al., 2018), its habitat and web construction resemble those of Arachnocampa spp, which live in similar humid cave environments. As previously suggested (Amaral et al., 2021), web construction and silk production could be phenotypic characteristics that may have evolved independently in Keroplatidae, similarly to bioluminescence.
